# Acromion bone block transfer with preserving coracoacromial ligament for surgical treatment of anterior instability novel method (Reverse Latarjet)

**DOI:** 10.1016/j.xrrt.2025.100641

**Published:** 2025-12-12

**Authors:** Süleyman Semih Dedeoğlu, Mustafa Buğra Ayaz, Yavuz Şahbat, Yasin Güler, Ahmet Keskin, Yunus İmren, Bülent Karslıoğlu

**Affiliations:** a Department of Orthopedics and Traumatology, Istinye University Faculty of Medicine, Liv Hospital, Istanbul, Turkey; b Department of Orthopedics and Traumatology, University of Health Sciences, Baltalimanı Bone Diseases Training and Researh Hospital, Istanbul, Turkey; c Department of Orthopedics and Traumatology, Istinye University Bahcesehir Liv Hospital, Istanbul, Turkey

**Keywords:** Latarjet, Reverse Latarjet, Bone block, Coraco-acromial ligament, Shoulder, Dislocation

Recurrent anterior shoulder dislocations can lead to an anterior glenoid bone defect.^4^^,^^19^ As the size of the glenoid defect increases, the success of Bankart surgery decreases. For large glenoid bone defects associated with recurrent traumatic anterior shoulder dislocations, bone block procedures are often preferred for treatment and management.^8^^,^^9^^,^^22^ Among the several described bone grafting procedures, the one most commonly used is the Latarjet procedure, which involves the transfer of the ipsilateral coracoid process with the conjoint tendon onto the anterior glenoid, followed by surgical techniques involving either the transfer of autograft including iliac crest, contralateral femoral condyle or clavicle, or allograft such as distal tibia, proximal tibia, distal femur, iliac crest, and femoral head.^10^^,^^17^ The stabilizing effect of the Latarjet open procedure on the shoulder is explained by 3 mechanisms: The “bone blocking effect”, which restores glenoid bone loss; the “sling effect”, in which the conjugated tendon limits anterior translation in abduction and external rotation; the hammock effect of the inferior part of the subscapular tendon; and the “ligament effect”, in which the remnant of the coracoacromial ligament (CAL) is used to reconnect the medial capsule.^1^^,^^2^^,^^15^^,^^16^ The Eden-Hybinette procedure has been described as open or arthroscopic transfer of a tricortical iliac bone autograft to address glenoid bone defects. Classic indications for this method include severe glenoid bone loss (>40%), Latarjet or distal tibial allograft failure, or abnormal coracoid morphology.^18^

The Latarjet procedure offers several surgical advantages, including a single incision, graft harvesting from the ipsilateral coracoid, and transfer over a short distance.^2^ While the Latarjet procedure has advantages, such as a combined mechanism for anterior shoulder stability, its complication rates of 15%-30% remain a concern. Well-known complications include graft fracture or malposition, neurovascular injury, and shoulder motion limitation for short term; medium-term issues such as nonunion and screw breakage; and long-term problems like graft osteolysis and glenohumeral arthrosis.^11^^,^^12^ Studies have reported a failure rate of 11% attributed to graft osteolysis or fracture.^28^ The Latarjet procedure can complicate subsequent revision surgeries, which is another drawback.^26^

This surgical technique introduces a novel acromial graft-derived method aimed at reducing donor site morbidity and minimizing complications related to graft osteolysis and nonunion at the glenoid. This procedure was named 'Reverse Latarjet’, since the procedure preserves the CAL's coracoacromial footprint and utilizes an acromial graft from the acromial footprint site.

## Indications

This technique is indicated for patients with large glenoid bone defect in whom Bankart surgery has failed. Although the initial indication was defined for revision cases after failed Bankart repair, the anatomical feasibility suggests potential applicability in primary large glenoid defects as well.

## Study design

Ethics committee approval was granted from Metin Sabanci Baltalimani Bone Diseases Training and Research Hospital on June 26, 2024 (decision no: 156). Computed tomography (CT) and shoulder cadaveric specimen were used to demonstrate the surgical technique. This is a three-dimensional (3D) computer-aided design (CAD) model reconstructed from Digital Imaging and Communications in Medicine (DICOM) data of an 18-year-old male patient who presented to the emergency department with trauma but whose right shoulder CT showed no major osseous pathology and a right-sided shoulder specimen of a 52-year-old male fresh frozen cadaver.

## Materials

Eighteen-year-old patient's right shoulder CT DICOM data were accessed through ExtremePACS (ExtremePACS, Çankaya/Ankara, Turkey) server. The DICOM images were then imported into Materialise Mimics Medical (version 21.0; Materialise, Leuven, Belgium). The scapula and clavicle images were isolated and 3D reconstructed. The resulting CAD model with Standard for the Exchange of Product extension was imported into SolidWorks Premium 2021 (version SP4.1; Dassault Systèmes SolidWorks Corp., Waltham, MA, USA) to investigate the feasibility of graft transferring while preserving ligament integrity. In addition, the CAD model was subjected to computer-aided manufacturing procedures and printed on the selective laser sintering 3D printer to examine the details of the technique and to ensure that the digital measurements were consistent with the rough manual measurements.

The fresh frozen cadaveric specimen used in this study was a 52-year-old male with no previous surgical history, donated to a tissue bank for medical research and obtained by our institution after arrangements were made through the Department of Anatomy, University of Health Sciences. The cadaveric right shoulder specimen was stored at 20°C and thawed at room temperature for 24 hours before preparation. The humerus of the cadaveric shoulder specimen was transected 20 cm proximal to the diaphysis, preserving all soft tissues within 15 cm of the glenohumeral joint medially. The specimen was clamped with a vise over the medial aspect of the scapula. The cadaveric specimen was then approached through a deltopectoral incision. As a result of the nature of the descriptive study, the humeral head was resected with a saw and most of the deltoid, including its anterior and mid portion, was excised. The acromion was largely stripped of soft tissues from the anterior viewpoint, and its anterior and anterolateral (AL) portions were identified. Similarly, the anterior shoulder muscles and surrounding soft tissues, including the biceps and subscapularis tendons, were removed to reduce visual obstruction of the anterior glenoid.

## Methods

The preliminary study analyzed the feasibility of transferring an acromial-derived bone graft while preserving the CAL structure using SolidWorks 2021 software on a 3D reconstruction of scapula. The glenoid anatomical structure of the model was categorized as pear-shaped and the variation of the acromion anatomical structure was categorized as type 1. Based on this analysis, considering the “V-shaped” or “Y-shaped” anatomical variations of the CAL structure, 3 footprint points anteromedial, AL, and posterior (P) were identified on the acromion, along with their corresponding projections onto the coracoid process. Subsequently, 3 points were defined on the anterior rim of the glenoid to predict the placement of the acromial graft after transfer.

A comparative analysis was performed between the 3 coraco-acromial (CA) distances between the original donor site of the acromial bone graft and the coracoid with the 3 coraco-glenoid (CG) distances measured between the same points on the coracoid and the predetermined points on the glenoid. To clarify the measurement protocol, 3 reference points were identified on the CAL footprint on the acromion—anteromedial, AL, and P—and their corresponding points on the coracoid attachment were defined. The CA distances were measured between each acromial and coracoid point pair, while the CG distances were measured between the same coracoid points and the corresponding predicted glenoid sites after graft transfer. This simplified description highlights how the spatial relationship between the acromion, coracoid, and glenoid was used to evaluate the feasibility of CAL preservation.

**Figure 1:**
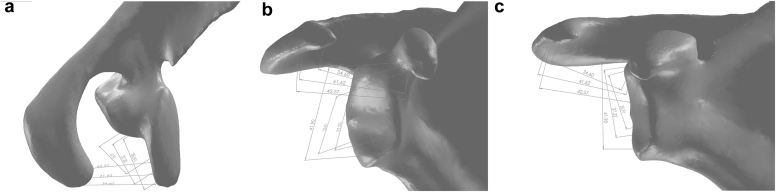
The landmarks of the coracoacromial ligament (CAL) on the acromion and coracoid were manually marked and 3 lengths examining the coraco-acromial (CA) distance in the coronal plane were defined (**a-c**). The distance between the most anterior point on the acromion footprint and the point marked anteromedial (AM) to the coracoid was defined as anterior distance and measured as 34.6 mm. The intermediate distance was defined as the anterolateral (AL) on the acromion footprint to the midpoint on the coracoid footprint and was measured as 41.6 mm. The posterior distance between the most posterior point on the acromion footprint of the CAL and the most posterior point on the attachment site on the coracoid was defined as the posterior distance and measured as 42.6 mm. After the graft was rotated 90° and transferred to the anteroinferior aspect of the glenoid, the new locations on the glenoid that were predicted to correspond to these 3 distances were determined so that the points determined on the coracoid remained the same, and 3 parameters indicating the coraco-glenoid (CG) distance were defined. The most anterior point on the acromion was positioned superior to the anteroinferior glenoid landmark, the midpoint of the acromion was positioned at the midpoint of the anteroinferior glenoid landmark and the most posterior point of the acromion was positioned at the inferior point of the anteroinferior glenoid landmark. As a result of this placement, the anterior distance was defined as the upper distance in its new position and measured 35.9 mm, the intermediate distance was defined as the middle distance in its new position and measured 37.2 mm, and the posterior distance was defined as the lower distance in its new position and measured 41.9 mm. Even when these measured distances included the margin of error, it was determined that the ligament would be positioned without the need for extra loosening or procedures to increase ligament tension after transfer.

In the initial evaluation of the CAD model on SolidWorks software, the glenoid anatomical structure of the model was categorized as pear-shaped and the variation of the acromion anatomical structure was categorized as type 1. As a result of manual measurements made on the model printed on a 3D printer, it was determined that the transfer of acromial bone as a graft while preserving the CAL was anatomically favorable. Measurements were made on the 3D reconstructed CAD model using SolidWorks Premium 2021 SP4.1 software (Fig. 1).

**Figure 2:**
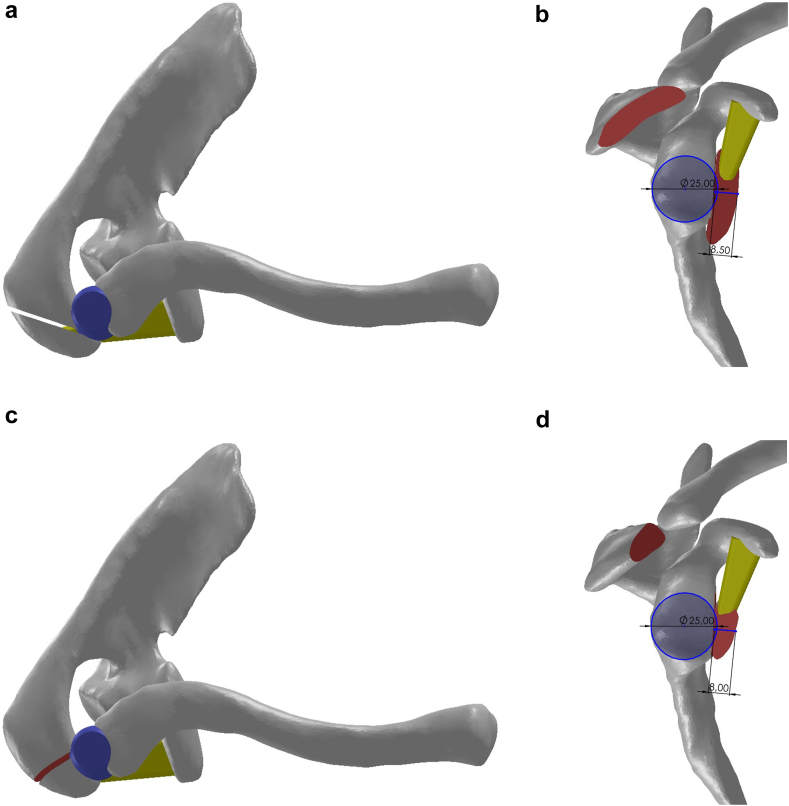
The patient had a glenoid length of 40 mm and the glenoid diameter was approximately 26 mm. A 1 mm bone incision was made to create a flat surface at the transplant recipient site anterior to the glenoid and the diameter of the new surface was measured as 25 mm. The illustration of the models after glenoid reconstruction using both methods is as shown in Figure. In reconstructions performed with the open (AC sparing) technique (**a** and **b**), the average graft thickness was observed to be 8.5 mm and the graft length was longer, whereas in the closed (AC sacrificed) technique (**c** and **d**), the graft length was found to be approximately 8 mm, which was shorter than that in the AC sparing technique. With these results, it can be concluded that open reconstruction has a defect reconstruction potential of approximately 33% and closed reconstruction has a defect reconstruction potential of approximately 30%. *AC*, acromioclavicular.

After the measurements, 2 different osteotomy techniques were defined on the 3D anatomical CAD model according to whether the acromioclavicular (AC) joint was preserved or not. The first method was described as open or 'AC sparing’ and the second method was described as closed or 'AC sacrificed’. The open method is so named because it is not conducive to arthroscopic surgery and the cadaveric study is based on this technique. Two different study models were created to evaluate graft placement after incisions to create defects in the donor site on the acromion and the recipient site on the glenoid for both techniques. Although the bones on the anatomical model were obtained exactly from the CT data, the drawings corresponding to the ligaments and joint capsule were visualized for demonstrative purposes (Fig. 2) Video 1.

**Figure 3:**
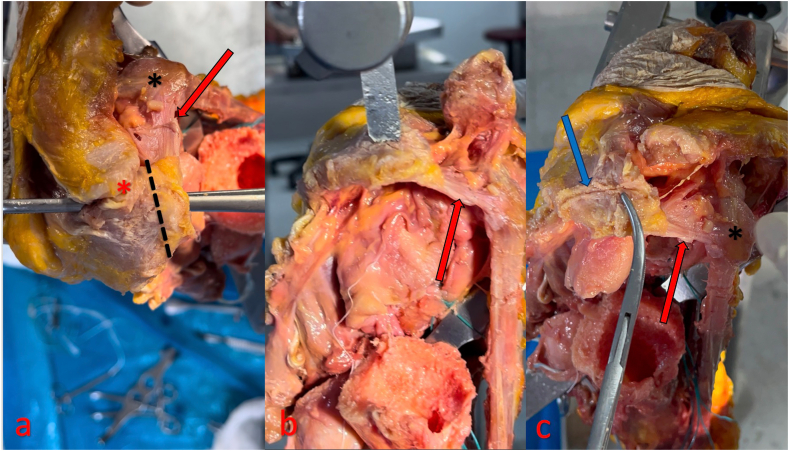
In this cadaveric study, the AC joint was dissected, the humeral head was incised, and the subscapularis tendon was lifted from the humerus and opened through a transverse incision to enhance the anatomical field of view following an extended deltopectoral approach. After removing all soft tissues from the acromion, a bone graft measuring between 4 mm and 11 mm in width was osteotomized from the donor site using a fine saw. Following the incision, it was demonstrated that the CAL structure, along with its attachment site and the AC joint, could be completely preserved. Neither a defect was created in the anterior glenoid nor was decortication performed. The graft was placed in a lying-down position and fixed with 2 Kirschner wires. The *red star* indicates the AC joint, and the *black star* indicates the coracoid process. The *red arrows* indicate the CAL, while the *blue arrow* marks the osteotomy line. *AC*, acromioclavicular; *CAL*, coracoacromial ligament.

**Figure 4:**
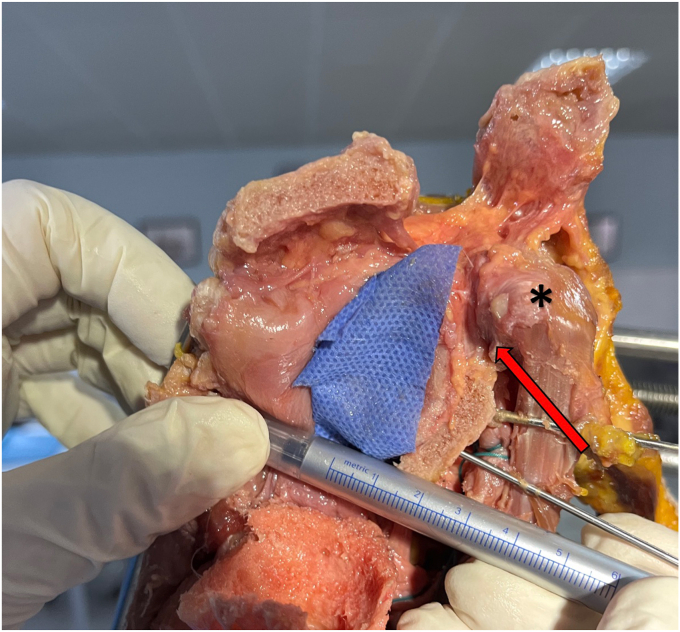
Manual measurements using a ruler determined the maximum glenoid width to be 25 mm, while the graft thickness averaged 8 mm, maintaining a homogeneous structure at nearly all levels. An anatomical cadaveric study demonstrated that acromial transfer can restore up to 32% of the glenoid bone surface. The *red arrows* indicate the CAL, while the *black star* marks the coracoid process. During cadaveric transfer, the graft was advanced between the supraspinatus and subscapularis planes toward the anterior glenoid rim. *CAL*, coracoacromial ligament.

Preliminary studies suggest that acromial bone graft transfer can be performed with preservation of the CAL.^3^ In order to support this technique and to include the steps associated with tissue dissection in this study, an ‘Open’ or ‘AC Sparing’ method was used on a cadaveric right shoulder specimen (Figs. 3 and 4). Table I summarizes pearls and pitfalls.

**Table I:** Pearls and pitfalls.

Step	Pearls & pitfalls
Preoperative planning	Computed tomography for distance measurement facilitates planning. Measure defect rate, good choice for defects up to 33%
Surgical approach	Both techniques can perform in the beach chair position.
Equipment	Fine saw to avoid bone loss
Fixation	
AC sparing	Endo-button
AC sacrificed	Screw fixation, screw and drill diameter can be modified.
Postoperative period	Simple arm sling

*AC*, acromioclavicular.

## Discussion

This technique aims to reconstruct anterior glenoid bone defects caused by anterior shoulder instability. Our study was designed in 2 phases: (1) measurements on digital 3D reconstruction images and (2) performing the procedure on a cadaver. The technique, defined as the reverse Latarjet, appears to be a biomechanically promising option for anterior glenoid defects. Manual and digital measurements on a 3D scapula anatomical model obtained from an 18-year-old male patient by CAD/computer-aided manufacturing method showed that the CA and CG distances were compatible with each other. Kesmezacar et al presented that the shape, length and thickness of the CAL anatomy may be affected by age, acromion type and cuff degeneration.^13^ If the CA distance is shorter than the CG distance, certain techniques could be used to compensate for the length difference. First of all, viscoelastic ligaments can be stretched intraoperatively even without the need for loosening from surrounding tissues. Secondly, additional length can be achieved by loosening the posterior component or portion of the CAL's coracoid attachment site. This is particularly important in its common Y-shaped variation, where loosening over the coracoid footprint becomes most necessary.^13^ In addition, positioning the bony component of the graft more superiorly may also be considered as a fixation preference if deemed appropriate. Measuring the CA and CG distances with preoperative CT in patients in whom the current procedure is planned will reduce potential challenges.^5^ Moreover, the length of the bone component of the graft in the open (AC sparing) method will allow the length difference to be tolerated in many respects. If the length of the CAL is judged to be shorter than the preferred level, opting for the open method may be advantageous. While the surgical exposure and portal orientation in the closed (AC sacrificed) method resemble those used in the arthroscopic distal clavicle resection (Mumford procedure), the graft harvesting process is technically more demanding.^23^ Upon further analysis of the technique, it should be noted that it can be performed using the same portals and surgical instruments as those employed in Bankart surgery, without necessitating advanced surgical expertise. The use of posterior drill guides and graft drill guides is recommended in this technique as it is expected to facilitate the procedure and reduce the risk of complications.

In the current literature, bone block procedures for glenoid defects are recommended for patients with glenoid bone loss greater than 15% or 25% (subcritical and critical, respectively), or patients classified in groups III and IV of the Di Giacomo algorithm, or patients with Instability Severity Index Score (ISI score) or Glenoid Track Instability Management Score greater than 4 points.^8^^,^^9^^,^^22^ Various bone graft options have been described in the literature, including the ipsilateral distal clavicle,^14^^,^^24^ the posterior aspect of the acromion^21^ and the AC articular aspect of the acromion,^27^ among which nonvascularized autografts are preferred.^14^^,^^21^^,^^24^^,^^27^ The most commonly used surgical technique in practice is the Latarjet procedure. Table II compares the Latarjet and reverse Latarjet procedures. The Latarjet procedure has undergone many modifications since its inception. The original Bristow procedure, described in a similar period to the Latarjet, has some fundamental differences with the same aim. Some of them can be classified under headings including osteotomy site, osteotomy method, tenotomy method, and fixation technique as well as the placement of the bone block. In the Bristow procedure, the resected base of the coracoid graft is fixed on the glenoid vault in the 'standing up’ position with a suture instead of fixation with a single screw in the 'lying down’ position with the inferior region of the coracoid on the glenoid dome as described in the Latarjet procedure.^6^ The Bristow technique, which subsequently underwent many modifications to the original Latarjet, is now referred to as Bristow-Latarjet in reference to bone procedures using the coracoid.^25^ It has been reported that resorption in the Latarjet procedure most commonly involves the superior and superficial part of the coracoid, but devascularization can be limited by reducing the pectoralis minor release from the tip of the coracoid process. Di Giacomo G. et al also reported that the bone block underwent significantly more osteolysis in patients without anterior glenoid bone defects compared to those with significant glenoid bone loss.^7^ The aim of the current study is to obtain the bone graft to be harvested from the acromion by preserving the CAL, including its attachment site, just as in the modified Weawer-Dunn-Chuinard procedure, and to transfer it to the recipient site together.^3^ Thus, the bone graft will be nourished through the CAL, which may help reduce the risk of graft osteolysis. The blood supply of the CAL structure originates from the branches of the thoracoacromial artery entering the CAL from the acromial and coracoid entheses. On the superior side, the CAL is continuous with the deltoid fascia where it adheres along the acromion, while near the coracoid it is usually divided into an AL band and a posteromedial band. These structures are often separated by a thin membrane, but 5 different variations are mentioned.^20^ Another expectation is that the CAL structure provides a suspension and ligament effect as well as supporting the nutrition of the bone graft. In addition, other graft alternatives are preserved, including coracoid procedures that can be performed in case of failure. However, the modified Weawer-Dunn-Chuinard procedure, which can be applied if needed in the treatment of AC injury that may develop later, is waived.

**Table II:** Advantages and disadvantages.

	Latarjet procedure	Reverse Latarjet
Revision	Iliac bone block or autograft	Latarjet procedure can be applied
Possible morbidity	Pectoralis minor weakness	Anterior deltoid weakness
	Conjoint tendon weakness	
Graft blood supply	Graft blood supply is available (conjoint tendon)	Graft blood supply is available (coracoacromial ligament)
AC joint	No pathology	No pathology (AC sparing procedure)
Mechanism	Bone blocking effect	Bone blocking effect
	Sling effect	Labrum augmentation effect
	Ligament effect	

*AC*, acromioclavicular.

The main concern of the authors regarding the description of the technique is the possible deltoid muscle origo injury if gentle dissection is not performed during the acromial bone incision. Gentle dissection of the incision segment may be necessary to avoid possible donor-site morbidity.

To optimize deltoid preservation, the anterior deltoid can be elevated subperiosteally from the acromion using a periosteal elevator, maintaining its bony origin and minimizing fiber disruption during exposure. This subperiosteal approach allows adequate visualization for graft harvesting while protecting the deltoid attachment. Further studies can be designed with possible deltoid muscle electromyography analysis once the clinical application of the technique is initiated. Another limitation of our study may be anatomical variations. The importance of anatomical variations in the Latarjet procedure is less due to the flexibility of the conjoint tendon, but in the presence of possible anatomical variations in the defined surgical technique, the position of the graft should be changed without disrupting the CAL circulation or the CAL should be loosened from the corocoid footprint.

Although the cadaveric phase of this study was performed through an open deltopectoral approach to ensure optimal visualization and measurement accuracy, the reverse Latarjet technique can be adapted for arthroscopic application using standard anterior and AL Bankart portals. The graft trajectory between the supraspinatus and subscapularis planes is compatible with arthroscopic visualization. An additional anterosuperior portal may further facilitate graft handling, orientation, and precise osteotomy using an arthroscopic saw. With the aid of posterior drill guides and cannulated fixation systems, the bone transfer can be performed safely and reproducibly. The authors plan to develop and validate a step-by-step arthroscopic protocol in a subsequent cadaveric phase prior to clinical implementation.

## Conclusion

In this study, the technique called “reverse Latarjet procedure” showed that acromion bone tissue is a potential graft that can be used in glenoid bone defects after anterior instability with preservation of CAL. With its bone block effect, more than 30% of bone loss may potentially be reconstructed. Furthermore, it is promising as a protective technique against graft lysis due to the perforating artery passing through the CAL. A future study by the authors will focus on presenting the clinical and radiologic outcomes of patients treated with the reverse Latarjet procedure.

## Disclaimers

Funding: No funding was disclosed by the authors.

Conflicts of interest: The authors, their immediate families, and any research foundation with which they are affiliated have not received any financial payments or other benefits from any commercial entity related to the subject of this article.

## References

[1] Bauer S., Collin P., Zumstein M.A., Neyton L., Blakeney W.G. (2023). Current concepts in chronic traumatic anterior shoulder instability. EFORT Open Rev.

[2] Boileau P., Mercier N., Roussanne Y., Thélu C.-É., Old J. (2010). Arthroscopic bankart-bristow-latarjet procedure: the development and early results of a safe and reproducible technique. Arthroscopy.

[3] Boileau P., Old J., Gastaud O., Brassart N., Roussanne Y. (2010). All-arthroscopic weaver-dunn-chuinard procedure with double-button fixation for chronic acromioclavicular joint dislocation. Arthroscopy.

[4] Boileau P., Villalba M., Héry J.-Y., Balg F., Ahrens P., Neyton L. (2006). Risk factors for recurrence of shoulder instability after arthroscopic Bankart repair. J Bone Joint Surg Am.

[5] Chahla J., Marchetti D.C., Moatshe G., Ferrari M.B., Sanchez G., Brady A.W. (2018). Quantitative assessment of the coracoacromial and the coracoclavicular ligaments with 3-dimensional mapping of the coracoid process anatomy: a cadaveric study of surgically relevant structures. Arthroscopy.

[6] Cowling P., Akhtar M., Liow R. (2016). What is a Bristow-Latarjet procedure?: a review of the described operative techniques and outcomes. Bone Joint J.

[7] Di Giacomo G., de Gasperis N., Costantini A., De Vita A., Beccaglia M.A.R., Pouliart N. (2014). Does the presence of glenoid bone loss influence coracoid bone graft osteolysis after the Latarjet procedure? A computed tomography scan study in 2 groups of patients with and without glenoid bone loss. J Shoulder Elbow Surg.

[8] Di Giacomo G., Itoi E., Burkhart S.S. (2014). Evolving concept of bipolar bone loss and the hill-sachs lesion: from “engaging/non-engaging” lesion to “on-track/off-track” lesion. Arthroscopy.

[9] Di Giacomo G., Peebles L.A., Pugliese M., Dekker T.J., Golijanin P., Sanchez A. (2020). Glenoid track instability management score: radiographic modification of the instability severity index score. Arthroscopy.

[10] Gilat R., Haunschild E.D., Lavoie-Gagne O.Z., Tauro T.M., Knapik D.M., Fu M.C. (2021). Outcomes of the Latarjet procedure versus free bone block procedures for anterior shoulder instability: a systematic review and meta-analysis. Am J Sports Med.

[11] Griesser M.J., Harris J.D., McCoy B.W., Hussain W.M., Jones M.H., Bishop J.Y. (2013). Complications and re-operations after Bristow-Latarjet shoulder stabilization: a systematic review. J Shoulder Elbow Surg.

[12] Gupta A., Delaney R., Petkin K., Lafosse L. (2015). Complications of the Latarjet procedure. Curr Rev Musculoskelet Med.

[13] Kesmezacar H., Akgun I., Ogut T., Gokay S., Uzun I. (2008). The coracoacromial ligament: the morphology and relation to rotator cuff pathology. J Shoulder Elbow Surg.

[14] Kwapisz A., Fitzpatrick K., Cook J.B., Athwal G.S., Tokish J.M. (2018). Distal clavicular osteochondral autograft augmentation for glenoid bone loss: a comparison of radius of restoration versus Latarjet graft. Am J Sports Med.

[15] Lafosse L., Boyle S. (2010). Arthroscopic latarjet procedure. J Shoulder Elbow Surg.

[16] Lafosse L., Lejeune E., Bouchard A., Kakuda C., Gobezie R., Kochhar T. (2007). The arthroscopic Latarjet procedure for the treatment of anterior shoulder instability. Arthroscopy.

[17] Longo U.G., Loppini M., Rizzello G., Ciuffreda M., Maffulli N., Denaro V. (2014). Latarjet, Bristow, and eden-hybinette procedures for anterior shoulder dislocation: systematic review and quantitative synthesis of the literature. Arthroscopy.

[18] Lunn J.V., Castellano-Rosa J., Walch G. (2008). Recurrent anterior dislocation after the Latarjet procedure: outcome after revision using a modified eden-hybinette operation. J Shoulder Elbow Surg.

[19] Moya D., Aydin N., Yamamoto N., Simone J.P., Robles P.P., Tytherleigh-Strong G. (2021). Current concepts in anterior glenohumeral instability: diagnosis and treatment. SICOT J.

[20] Rothenberg A., Gasbarro G., Chlebeck J., Lin A. (2017). The coracoacromial ligament: anatomy, function, and clinical significance. Orthop J Sports Med.

[21] Sanchez M., Klouche S., Faivre B., Bauer T., Hardy P. (2017). Acromial J-bone graft on the acromion for surgical treatment of glenohumeral instability: an anatomical study. Shoulder Elbow.

[22] Shaha J.S., Cook J.B., Song D.J., Rowles D.J., Bottoni C.R., Shaha S.H. (2015). Redefining “critical” bone loss in shoulder instability: functional outcomes worsen with “subcritical” bone loss. Am J Sports Med.

[23] Snyder S.J., Banas M.P., Karzel R.P. (1995). The arthroscopic Mumford procedure: an analysis of results. Arthroscopy.

[24] Tokish J.M., Fitzpatrick K., Cook J.B., Mallon W.J. (2014). Arthroscopic distal clavicular autograft for treating shoulder instability with glenoid bone loss. Arthrosc Tech.

[25] Weaver J.K., Derkash R.S. (1994). Don't forget the Bristow-Latarjet procedure. Clin Orthop Relat Res.

[26] Willemot L.B., Elhassan B.T., Sperling J.W., Cofield R.H., Sánchez-Sotelo J. (2018). Arthroplasty for glenohumeral arthritis in shoulders with a previous Bristow or Latarjet procedure. J Shoulder Elbow Surg.

[27] Zhang J.A., Lam P., Beretov J., Murrell G.A. (2023). Acromion and distal clavicle grafts for arthroscopic glenoid reconstruction. J Clin Med.

[28] Zimmermann S.M., Scheyerer M.J., Farshad M., Catanzaro S., Rahm S., Gerber C. (2016). Long-term restoration of anterior shoulder stability: a retrospective analysis of arthroscopic Bankart repair versus open Latarjet procedure. J Bone Joint Surg Am.

